# Early diagnosis of melanoma: a randomized trial assessing the impact of the transmission of photographs taken with a smartphone from the general practitioner to the dermatologist on the time to dermatological consultation

**DOI:** 10.1186/s12913-024-11106-9

**Published:** 2024-05-24

**Authors:** Céline Bouton, Héloïse Schmeltz, Charlotte Lévèque, Aurélie Gaultier, Gaëlle Quereux, Brigitte Dreno, JM Nguyen, Cédric Rat

**Affiliations:** 1 Department of General Practice, Faculty of Medicine of Nantes, 1 rue G. Veil, Nantes Cedex 1, 44035 France; 2 Department of Epidemiology and Biostatistics, Nantes University Hospital, 85 rue Saint Jacques, Nantes Cedex 1, 44093 France; 3 National Institute for Medical Research – Unit, 1302 Team 2, Nantes, France; 4 Department of Dermatology, Nantes University Hospital, 1 place Alexis Ricordeau, Nantes Cedex 1, 44093 France

**Keywords:** Melanoma, Skin cancer, Early diagnosis, Primary care, General practitioners, Teledermatology, Smartphone, Mobile phone, Consultation delay, Patient care pathways

## Abstract

**Background:**

Difficulty obtaining a dermatological consultation is an obstacle to the early diagnosis of melanoma. On the one hand, patients survival depends on the lesion thickness at the time of diagnosis. On the other hand, dermatologists treat many patients with benign lesions. Optimizing patient care pathways is a major concern. The aim of the present study was to assess whether the e-mail transmission of photographs of suspected melanoma lesions between general practitioners (GPs) and dermatologists reduces the time to dermatological consultation for patients whose suspicious skin lesions ultimately require resection.

**Methods:**

We conducted a cluster-randomized controlled study in primary care involving 51 French GPs between April 2017 and August 2019. A total of 250 patients referred to a dermatologist for a suspected melanoma lesion were included GPs were randomized to either the smartphone arm or the usual care arm. In the smartphone arm, the GPs referred patients to the dermatologist by sending 2 photographs of the suspicious lesion using their smartphone. The dermatologist then had to set up an appointment at an appropriate time. In the usual care arm, GPs referred patients to a dermatologist according to their usual practice. The primary outcome was the time to dermatological consultation for patients whose lesion ultimately required resection.

**Results:**

57 GPs volunteered were randomized (27 to the smartphone arm, and 30 to the usual care arm). A total of 125 patients were included in each arm (mean age: 49.8 years; 53% women) and followed 8 months. Twenty-three dermatologists participated in the study. The time to dermatological consultation for patients whose suspicious skin lesion required resection was 56.5 days in the smartphone arm and 63.7 days in the usual care arm (mean adjusted time reduction: -18.5 days, 95% CI [-74.1;23.5], *p* = .53).

**Conclusions:**

The e-mail transmission of photographs from GPs to dermatologists did not improve the dermatological management of patients whose suspicious skin lesions ultimately required resection. Further research is needed to validate quality criteria that might be useful for tele-expertise in dermatology.

**Trial Registration:**

Registered on ClinicalTrials.gov under reference number *NCT03137511 (May 2, 2017)*.

**Supplementary Information:**

The online version contains supplementary material available at 10.1186/s12913-024-11106-9.

## Background

In 2020, melanoma was responsible for 57,043 deaths worldwide, and its annual incidence continues to increase [1]. Cutaneous melanoma has a good prognosis if it is diagnosed early in situ or when its Breslow index is less than 1 mm (leading to a 10-year survival rate greater than 91%) and no remote metastases are present [2]. In the early stages, cutaneous melanoma can be cured via surgical resection. On the contrary, the prognosis of melanoma is poor when it metastasizes or when it is diagnosed at a late stage [1, 3]. In total, the main prognostic factor at the time of diagnosis is tumour thickness [2].

None of the existing guidelines recommend systematic screening for melanoma. In France, early diagnosis of melanoma is based on the identification of high-risk populations as well as complete skin examinations allowing the identification of atypical nevic lesions [4]. In accordance with other international recommendations [5], the French authorities for health policy recommend that identification of a suspicious lesion should be followed by a dermatologist consultation within 3 weeks at most.

However, previous authors have reported that the time to consultation with a dermatologist is long in France. Millien et al. reported a mean time of 61 days in a French epidemiological study conducted in 2016–2017 [6]. Rogowska et al. suggested that informal direct exchanges between general practitioners (GPs) and dermatologists might reduce this time in cases of recent lesion progression [7].

One hypothesis for this reduction is that sending photographs taken via smartphone might reduce the time to obtain a consultation. While 23% of French GPs reported sometimes sending photographs to dermatologists, only 4% reported that they do so on a regular basis in current practice [8].

The main objective of the study was to assess whether the email transmission of photographs of suspected melanoma lesions between GPs and dermatologists reduces the time to dermatological consultation for patients with a suspicious lesion that ultimately requires resection.

The secondary objectives were to assess whether the transmission of photographs (1) reduced the proportion of noncompliant patients (those who did not consult a dermatologist after referral by their GP for a suspicious lesion) and (2) led to an inappropriate reduction in the time to consultation for patients with a suspicious lesion that did not ultimately require resection.

## Methods

### Design

An open-label, controlled, cluster-randomized study with two parallel arms was conducted in western France (the Loire-Atlantique and Vendée geographic areas) between April 2017 and August 2019. The study protocol was prepared according to the 2013 SPIRIT guidelines for clinical trial protocols [9].

### Participants

Information on the study was mailed to GPs belonging to the practice-based research network of the Nantes University General Practice Department in November 2016 (approximately 350 GPs). To participate, volunteer GPs were asked (1) to provide the name of the dermatologists to which they usually referred their patients; (2) to update their knowledge on melanoma screening during a meeting coordinated by the study team; and (3) to use a smartphone that could send photographs that were not compressed below 100 KB. They participated in a training session on melanoma screening proposed by the research team in which they were taught how to take photographs with their smartphone and what clinical information was needed in addition to the photographs.

Of the 68 dermatologists in the region, 23 were cited by the GPs as their usual correspondent and were contacted to enquire whether they agreed to participate. Volunteer dermatologists were asked to provide their email addresses and had to consent to receive photographs of suspected melanoma lesions. Of the 350 GPs contacted, 57 participated in the study: 59 initially volunteered, but 2 ultimately withdrew their consent.

Patients had to meet the following inclusion criteria: (1) aged 18 years or older; (2) consultation with a GP participating in the study; (3) presentation with a suspected melanoma lesion requiring a referral to a dermatologist according to the GP; (4) referral to a dermatologist who agreed to participate in the study; (5) affiliation with a health insurance program; and (6) provision of written informed consent.

### Smartphone arm and usual care arm

The GPs’ medical practices were randomized to either the smartphone arm or the usual care arm.

In the smartphone arm, the GPs were required to (1) collect clinical patient information, including age, sex, risk factors for melanoma (personal and family history of skin cancer, phototype, number of *naevi* on both arms > 20, presence of freckles, stay in a country with strong sunshine for more than a year, history of severe sunburn in childhood), and descriptive features of the lesion (based on the ABCDE criteria) [10]; (2) take two photographs of the suspected melanoma lesion with their smartphone, with the first photograph showing 20 cm around the lesion and the second photograph focusing on the lesion with a 2-cm integument area on either side; (3) send the two photographs of the suspicious lesion to the dermatologist by e-mail, along with relevant clinical information and the patient’s initials; and (4) call the dermatologist to inform him or her of the appointment request and provide the patient’s identity and contact details.

In the usual care arm, the GPs were asked to refer patients with a suspected melanoma lesion to a dermatologist without changing their usual practice.

Patients (smartphone arm and usual care arm) were informed about the study and invited to participate by their GPs during the consultation. Patients could either object or were asked to sign a written consent form before being allocated to the intervention smartphone group or usual care group.

### Randomization to the smartphone arm or usual care arm

The design was a cluster-randomized study with two parallel arms. To avoid contamination bias, the randomization clusters were medical practices rather than the GPs themselves; thus, two GPs working in the same practice were assigned to the same arm. The randomization was stratified according to the geographic area (either Loire-Atlantique or Vendée). The allocation ratio was 1:1.

The random allocation sequence, as well as the assignment of the participants to interventions, were generated by the methodologist of the research team.

### Endpoints and data collection

The primary endpoint was the consultation delay for patients whose suspicious skin lesion ultimately required resection. Skin lesions requiring resection were identified retrospectively after a skin examination according to the conclusion of the dermatologist.

The time between the GP consultation and the dermatologist consultation was calculated using the date of the GP consultation (as stated in the referring letter written by the GP) and the date of the dermatologist consultation (as stated in the response letter written by the dermatologist).

The secondary endpoints were (1) the proportion of noncompliant patients, i.e., patients referred to the dermatologist by their GP who had not consulted the dermatologist at the end of the study (August 1st, 2019), and (2) the time between the GP consultation and the dermatologist consultation for patients whose suspicious lesion did not require resection.

The following data were collected to characterize the GPs: age, sex, practice location, and distance (in km) between the medical practice of the GP and the practice of the nearest dermatologist. The following clinical data were collected to characterize the patients: age, sex, and risk factors for melanoma (personal and family history of skin cancer, phototype, number of naevi on both arms > 20, presence of freckles, stay in a country with strong sunshine for more than a year, and history of severe sunburn in childhood) [11].

Patients were included by the GPs between April 2017 and November 2018. The patients were then followed up until August 2019, i.e., 8 months after the end of the inclusion period. The data were collected by the study research team between February 1, 2018, and August 1, 2019, directly at the GPs’ medical practices.

### Statistical analysis

The calculation of the number of subjects needed was based on the prospective follow-up of the COPARIME cohort (consisting of 3,976 patients at high risk of melanoma), which revealed that for 149 patients referred for a suspected melanoma lesion that was ultimately resected, the mean time to consultation with the dermatologist was 95 days, with a variance of 156 days [10]. We hypothesized that this time could be shortened to 21 days due to the email transmission of smartphone photographs in the intervention arm. With an alpha risk of 5% in a two-sided situation and a power of 90% (beta risk of 10%), 532 patients had to be included.

The statistical unit was the patient. A modified intention-to-treat (mITT) analysis was performed; patients who did not meet all the inclusion criteria at the time of the analysis were excluded.

The statistical analysis of the primary endpoint was performed using a generalized linear mixed model with a random effect (GP’s medical practice). The analysis was adjusted for the geographic area and the distance between the GP’s practice and the dermatologist as fixed effects and for the GP’s medical practice as a random effect.

For the secondary endpoints, the proportions of noncompliant patients in both arms were also compared using a generalized linear mixed model according to the same strategy as for the primary endpoint. An analysis of the time between the GP consultation and the dermatologist consultation for patients whose suspicious lesion was ultimately not resected was performed using the same strategy.

The two-sided significance threshold was set to 5%. All analyses were performed using R 3.6.0 software (R Foundation for Statistical Computing, Vienna, Austria).

### Ethics approval and consent to participate

The OASE MELANOME protocol is in the field of Methodology Reference MR003, to which Nantes University Hospital conforms. Participation by GPs and dermatologists was voluntary and recorded in the form of written consent. The GPs proposed participation to their patients. Patients were asked to sign a written consent form to allow their data to be used for research purposes. This study involved professionals and human participants and was approved by the Ethics Committee of the National College of Teaching General Practitioners (Paris, France) (No. 1,061,721).

## Results

### GPs and patients

**Fig. 1:**
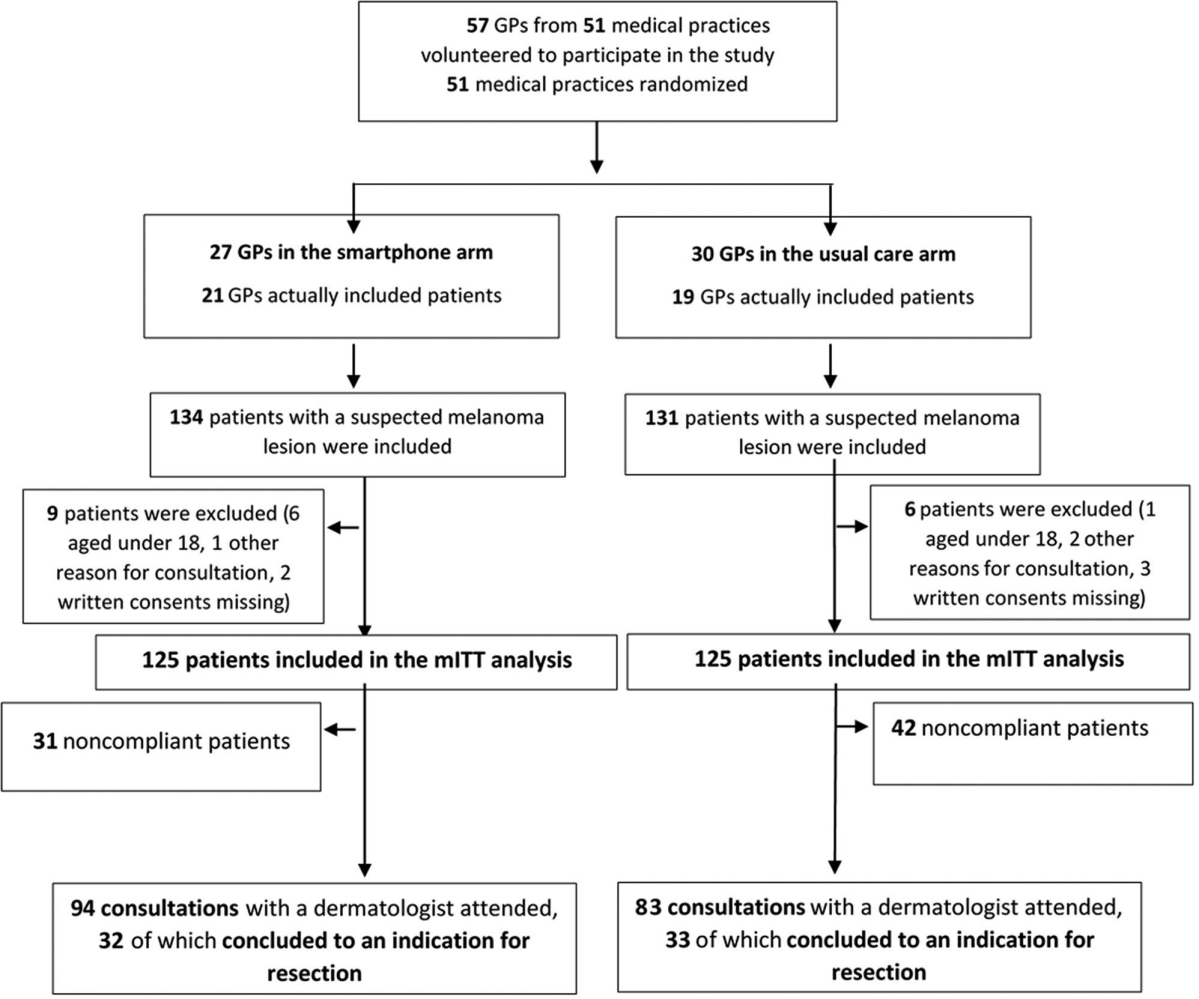
Flowchart of the study

Of the 350 GPs contacted, 57 GPs belonging to 51 medical practices volunteered to participate in the study and were randomized, as shown in the flow chart in Fig. 1: 27 were assigned to the smartphone arm, and 30 were assigned to the usual care arm. In the smartphone arm, the mean age of the GPs was 48.3 years, 48.1% (13/27) were women, and 11.1% (3/27) worked in rural areas. The mean distance between the GP practice and the nearest participating dermatologist was 13.4 km. In the usual care arm, the mean age of the GPs was 48.6 years, 46.7% (14/30) were women, and 3.3% (1/30) worked in rural areas. The mean distance between the GP practice and the nearest participating dermatologist was 10.2 km.

Among these 57 GPs, only 40 actually included patients in the study: 21 GPs in the smartphone arm and 19 GPs in the usual care arm.

Twenty-three dermatologists were contacted and participated in the study; 69.6% (16/23) were self-employed, whereas the other were salaried in a hospital. Additionally, 73.9% (17/23) were women.

Between April 2017 and November 2018, 265 patients were included: 134 in the smartphone arm and 131 in the usual care arm. The follow-up period lasted until August 1st, 2019. Seventeen patients were excluded from the mITT analysis: 9 in the smartphone arm (6 patients under 18 years of age, 1 patient referred to the dermatologist for a reason other than a suspicion of melanoma, and 2 patients not given their written consent) and 6 patients in the usual care arm (1 patient under 18 years of age, 2 patients referred to the dermatologist for a reason other than a suspicion of melanoma, and 3 patients not given their written consent). There were no missing data regarding the primary endpoint. The corresponding data are reported in Fig. 1 (flowchart of the study).

A total of 250 patients were included in the mITT analysis: 125 patients in the smartphone arm and 125 patients in the usual care arm. Patient demographics are reported in Table 1.

**Table 1:** Patient demographics

	Smartphone arm *N* = 125		Usual care arm *N* = 125		Total *N* = 250	
	n; *%*	Mean; (Standard deviation)	n, *%*	Mean; (Standard deviation)	n, *%*	Mean; (Standard deviation)
**Sex**						
Women	63; *50.4*		68; *55.7*		131; *53.0*	
Men	62; *49.6*		54; *42.3*		116; *47.0*	
Unknow	-		3		3	
**Age**						
		53.3; (17.9)		45.8; (16.5)		49.8; (17.6)
18–39	34; *27.2*		48; *42.5*		82; *34.5*	
40–59	46; *36.8*		39; *34.5*		85; *35.7*	
60–79	37; *29.6*		25; *22.1*		62; *26.1*	
≥80	8; *6.4*		1; *0.9*		9; *3.8*	
Unknow	-		12		12	
**History of melanoma**						
Personal	4; *3.2*		2; *1.6*		6; *2.4*	
Family	9; *7.2*		3; *2.4*		12; *4.8*	
**Distance “GP practice - dermatologist practice” (km, N = 57)**		13.4; (12.6)		10.2; (9.2)		11.7; (11.0)

### Time between the GP consultation and the dermatologist consultation for patients with a suspected melanoma lesion requiring resection

Among the 250 patients included in the mITT analysis, 65 required resection of their skin lesion: 32/125 in the smartphone arm and 33/125 in the usual care arm.

The mean time to dermatologist consultation for patients whose suspicious skin lesions required resection was 56.5 days in the smartphone arm and 63.7 days in the usual care arm. The adjusted mean difference was − 18.7 days (95% CI [-74.1;23.5], *p* = .53) (Table 2).

**Table 2:** The time to dermatologist consultation for patients with a lesion requiring resection

	Smartphone arm *N* = 32 patients Mean (Standard Deviation)	Usual care arm *N* = 33 patients Mean (Standard Deviation)	Adjusted difference [95% CI]	Adjusted *p* value
**Time to consultation** (days)	56.5 *(98.6)*	63.7 *(89.9)*	***-18.7 [-74.1;23.5]***	**0.53**

### Secondary outcomes

#### Patient compliance

The proportion of patients who did not consult a dermatologist after referral was 24.8% (31/125) in the smartphone arm *and* 33.6% (42/125) in the usual care arm (OR = 1.73, 95% CI [0.88;3.50], *P* = .10) (Table 3).

**Table 3:** Proportion of noncompliant patients (those who did not consult a dermatologist after referral)

	Smartphone arm *N* = 125 patients *n*; %	Usual care arm *N* = 125 patients *n*; %	Adjusted OR [95% CI]	Adjusted *p* value
**Noncompliant patients**	31 *(24.8)*	42 *(33.6)*	***1.73 [0.88;3.50]***	**0.10**

Time between the GP consultation and the dermatologist consultation for patients with a lesion that ultimately did not require resection

Among the 250 patients included in the mITT analysis, 112 attended a dermatologist consultation with a lesion that ultimately did not require skin resection: 62/125 in the smartphone arm and 50/125 in the usual care arm.

The mean time between the GP consultation and the dermatologist consultation for patients whose suspicious lesion did not require resection was 37.2 days in the smartphone arm and 74.4 days in the usual care arm. The adjusted mean difference was − 42.2 days (95% CI [-78.3;-6.1], *p* = .03) (Table 4).

**Table 4:** The time to dermatologist consultation for patients with a lesion that did not require resection

	Smartphone arm *N* = 62 Mean (Standard Deviation)	Usual care arm *N* = 50 Mean (Standard Deviation)	Adjusted difference [95% CI]	Adjusted *p* value
**Time to consultation** (days)	37.2 *(55.8)*	74.4 *(111.8)*	***-42.2 [-78.3;-6.1]***	**0.03**

## Discussion

### Main results

In our study, the email transmission of photographs of suspected melanoma lesions taken with a smartphone by the GP to the dermatologist did not lead to a significant optimization of the patient care pathway. Moreover, this approach did not shorten the time to dermatological consultation among patients whose lesions ultimately required resection. The transmission of photographs taken by the GP to the dermatologist and direct notification of the appointment to the patients did not significantly improve patient compliance with the dermatologist consultation compared to usual practice. In our study, the intervention specifically reduced the time to dermatological consultation for patients with suspected melanoma lesions that did not ultimately require resection.

### Strengths and limitations

This study has several strengths. First, this work is original, as there is a lack of international data about the management of patient care pathways among suspected melanoma cases. The impact of sending photographs taken with a smartphone has not been assessed in previous studies. Based on a previous review, our study appears to be the first to evaluate the impact of sending photographs taken with a smartphone by a GP to a dermatologist on the time to dermatologist consultation for suspected melanoma lesions [13, 14]. A second strength is the robustness of the study design, as it was a controlled, cluster randomized study. Third, our study was grounded in real daily practice: our control group was subjected to usual practices in primary care; the patient population included was representative of patients who consulted GPs, allowing high reproducibility [11]; and the implementation of the intervention did not result in any additional costs since the GPs were already equipped with Information Technology media (IT media) and used their own smartphones to take photographs. All the criteria collected by the GPs were well known to them, as they were used in daily practice and mentioned in recommendations and training courses.

Another strength of the study was the choice of the primary endpoint, defined here as the time to consultation for patients with a suspected melanoma lesion. This hypothesis was clinically relevant since it reflects the time to consultation for all pigmented lesions that the dermatologist had to resect (skin melanomas, benign lesions, other cancerous lesions, etc.) and not only the time to consultation for confirmed melanomas. Dermatologists were not asked to make a diagnosis based on the photographs. Indeed, focusing on optimization of the care pathway is a major issue, and various authors have reported difficulties in making a diagnosis based on photographs [12, 13].

This study has several limitations. The first limitation is the lack of statistical power due to the small number of patients included, which was half of the expected number; 250 patients were included, while an expected number of 532 patients was needed. The number of patients required was not reached despite a 13-month extension of the inclusion period. The inclusion period could not be extended for a longer period due to administrative reasons. The funder did not allow extending the period of expenses.

A second limitation is that we did not collect data about the GPs’ practices in the usual care arm. The participating GPs of both arms were volunteers, so they were very likely to be interested in and involved in melanoma screening. Thus, it is likely that they already directly intervened to hasten the dermatologist appointment (for example, by making phone calls), even in the usual care arm.

A final limitation might be that participating in the study required computer equipment and a smartphone. On the one hand, the use of internet media functionalities was reported to be limited in France compared with other countries [14]. On the other hand, smartphones have been widely used in recent years in private and in professional settings. The French data from Rogowska et al.’s study showed that only a small number of GPs sent photos to dermatologists, which may be outdated [7], and numerous applications have been developed in recent years. A question might be whether the participation rate of GPs should be considered as low. In primary care, particularly in France, GPs are not very used to take part in research activities. Among a network of 350 GPs, the total of 57 (16%) who agreed to become investigators should be considered as a fairly good ratio in this context.”

### Perspectives

In our study, the intervention specifically reduced the time to dermatological consultation for patients with suspected melanoma lesions that did not ultimately require resection. This result is consistent with those of the 2018 Cochrane teledermatology meta-analysis that investigated the remote assessment of skin lesions. When a remote assessment was performed, actions such as resection, referral to a specialist or follow-up were more often recommended than when decisions were made during a face-to-face consultation; this could accelerate the implementation of specialized management [15–17].

On the one hand, our results emphasize that too many benign lesions are referred to dermatologists; on the other hand, we concluded that dermatologists prefer to see patients face to face when they have the opportunity to be sure of their diagnosis. Qualitative studies could explore the barriers and facilitators of dermatologists in organizing this care pathway. Numerous leads could be explored to reduce the number of consultations for benign lesions and, therefore, the time taken to see a dermatologist: training GPs in dermatology and/or dermatoscopy [18], sending photographs, tele-expertizing procedures, decentralizing of dermatologists to interprofessional primary care teams, multidisciplinary meetings, using artificial intelligence when it becomes operational, etc.

As the French National Health Insurance System has recently decided to reimburse tele-expertise procedures, including the analysis of photographs of skin lesions by a dermatologist, increasing the number of studies that will provide high-quality standards for these practices is mandatory. An initial direction might be to duplicate this study in a larger population, allowing the inclusion of a greater number of lesions to be resected. In a systematic review of the literature, Trettel showed that worldwide, the main obstacles to the development of teledermatology were the lack of adequate secure technical infrastructure, the lack of reimbursement, local legal problems, the late involvement of healthcare systems and doctors’ lack of confidence in tele-expertise [19]. Fortunately, an increasing number of studies are have shown that teledermatology is reliable, cost effective and satisfying for patients and will gradually eliminate these obstacles.

Another direction would be to perform studies assessing teledermatoscopy, a technique that consists of sending dermoscopy photographs using a smartphone application or by email to a referent dermatologist. Interest in this technique has been demonstrated on several occasions. In 2015, Börve significantly reduced the time to consultation for histologically proven malignant lesions by sending teledermoscopy images directly to the dermatologist relative to delivering a letter to the patient, thereby reducing the mean time to obtain an appointment from more than 80 days to less than 2 days [20]. Although teledermoscopy might be relevant for optimizing patient care, its effectiveness in referring patients from primary care to a dermatologist has not yet been established. A previous review suggested that using teledermoscopy might be associated with greater tele-expertise performance [12]. Future studies should compare the improvement of patient care via teledermoscopy with that via clinical photography.

American studies on the stage of melanoma at the time of diagnosis or on the time between diagnosis and surgery suggest that not having health insurance is a factor in poorer care [21, 22]. These hypotheses should be confirmed by studies comparing prediagnosis times in primary care settings in countries with and without universal health insurance.

Even if our study results suggest that sending photographs of suspected melanoma according to the GP would not be discriminating, the use of this medium seems to be an expectation from the patients [23, 24], as it might improve their awareness of the disease.

## Conclusion

The e-mail transmission of photographs from GPs to dermatologists via smartphones did not improve the dermatological management of patients whose suspicious skin lesions ultimately required resection. However, further research is needed to validate the quality criteria for tele-expertise in dermatology, especially as this study was ultimately underpowered.

### Electronic supplementary material

Below is the link to the electronic supplementary material.

Supplementary Material 1

## Data Availability

The authors agree to share their data. Please contact the corresponding author should you require any of the results from this study.

## Abbreviations

GP: General practitioner
mITT: Modified intention-to-treat
IT media: Information Technology media
